# Noise annoyance through railway traffic - a case study

**DOI:** 10.1186/2052-336X-12-14

**Published:** 2014-01-08

**Authors:** Paulo Henrique Trombetta Zannin, Fernando Bunn

**Affiliations:** 1 Laboratory of Environmental and Industrial Acoustics and Acoustic Comfort, Federal University of Paraná, Curitiba, Paraná, Brazil

**Keywords:** Noise pollution, Railway noise, Noise measurements, Noise mapping, Environmental noise

## Abstract

This paper describes an assessment of noise caused by railway traffic in a large Latin American city. Measurements were taken of noise levels generated by trains passing through residential neighborhoods with and without blowing their horns. Noise maps were also calculated showing noise pollution generated by the train traffic. In addition - annoyance of the residents - affected by railway noise, was evaluated based on interviews. The measurements indicated that the noise levels generated by the passage of the train with its horn blowing are extremely high, clearly exceeding the daytime limits of equivalent sound pressure level - L_eq_ = 55 dB(A) - established by the municipal laws No 10.625 of the city of Curitiba. The L_eq_ = 45 dB (A) which is the limit for the night period also are exceeded during the passage of trains. The residents reported feeling affected by the noise generated by passing trains, which causes irritability, headaches, poor concentration and insomnia, and 88% of them claimed that nocturnal noise pollution is the most distressing. This study showed that the vast majority of residents surveyed, (69%) believe that the noise of the train can devalue their property.

## Introduction

Noise pollution today is no longer restricted to industrial environments but affects small, medium and large cities all over the world. It is a daily reality both in developed countries such as the United States and the European nations and in emerging countries such as India, China and Brazil.

Many sectors of society are affected by noise, particular which is generated by traffic. Traffic noise – *road*, *air*, and *railway* – causes discomfort and irritation, especially during activities that require attention and concentration [1-14].

Traffic noise is also a serious source of annoyance for people trying to rest and relax at home [15-18], particularly when it interferes with sleep, which is indispensable to human health, contributing to the degradation of quality-of-life [19-23].

Noise pollution in urban environments comes from numerous sources, e.g., sirens, loud music, neighbors, car and home alarms, religious temples, horns, motorcycles, trucks, passenger cars, buses, planes, trains, etc. [24-27].

**Figure 1:**
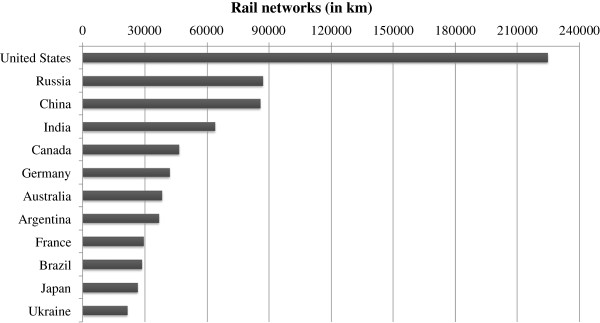
**Extent of rail networks in several countries (Adapted from [**[29]**]).**

Brazil’s rail network currently covers approximately 30,000 kilometers, and accounts for over 20% of the country’s freight transport [28]. Figure 1 compares the extent of the Brazilian rail network to that of other countries.

The Brazilian rail network is used primarily for transporting bulk commodities, such as soybeans, from the country’s producing regions to its shipping ports for export. The port of Paranaguá, situated in the state of Paraná in southern Brazil, is one of the main export outlets for the country’s agricultural production. In 2009, this shipping port handled 31.3 million tons of freight, of which approximately 8.6 million tons were transported by rail [30].

**Figure 2:**
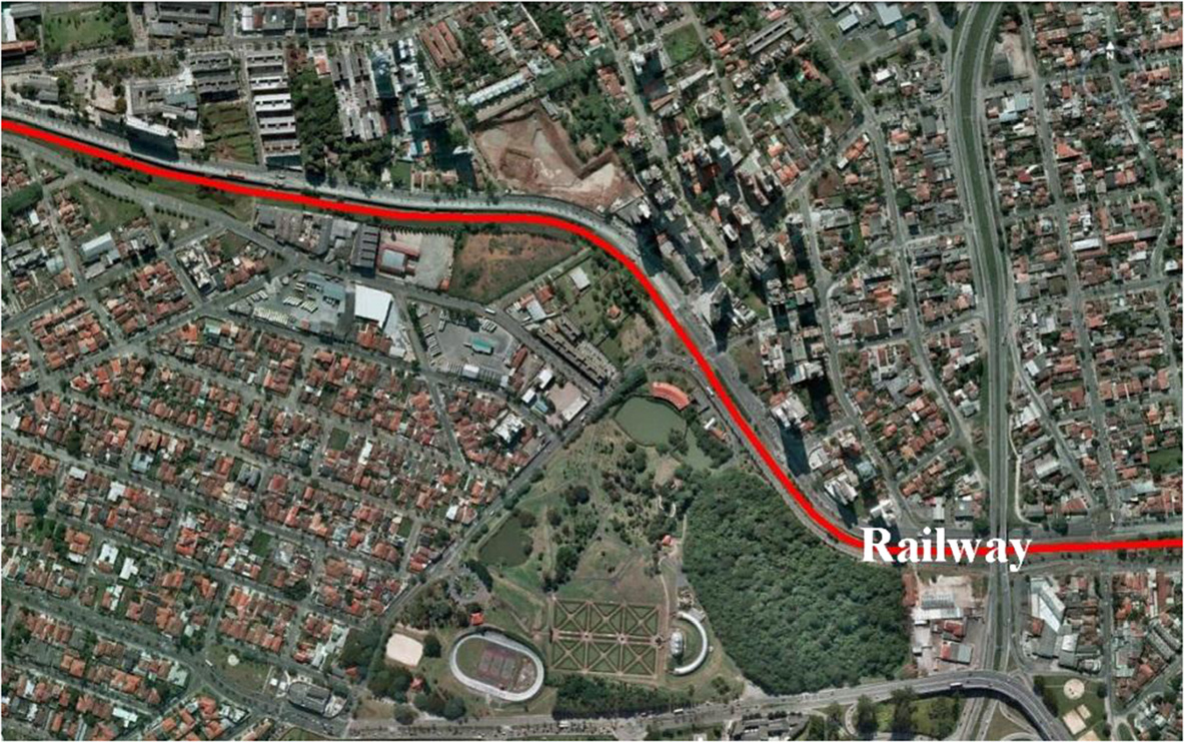
**A portion of the route covered by the railway line in the city of Curitiba.** Areas affected by train noise: Residential areas, Hospital and Public Park, Residential area under construction (five 12-story buildings).

The railway line linking the producer regions in the interior of the state of Paraná to this shipping port was built in the late 19^th^ century. On its route to the shipping port the railway line passes through Curitiba, the capital of the state of Paraná. The 319-year-old city of Curitiba is one of the oldest in Brazil, with a population of approximately 1.8 million. The stretch of railway line that runs through the city covers about 20 km. Figure 2 shows part of the route of the railway line through Curitiba.

**Figure 3:**
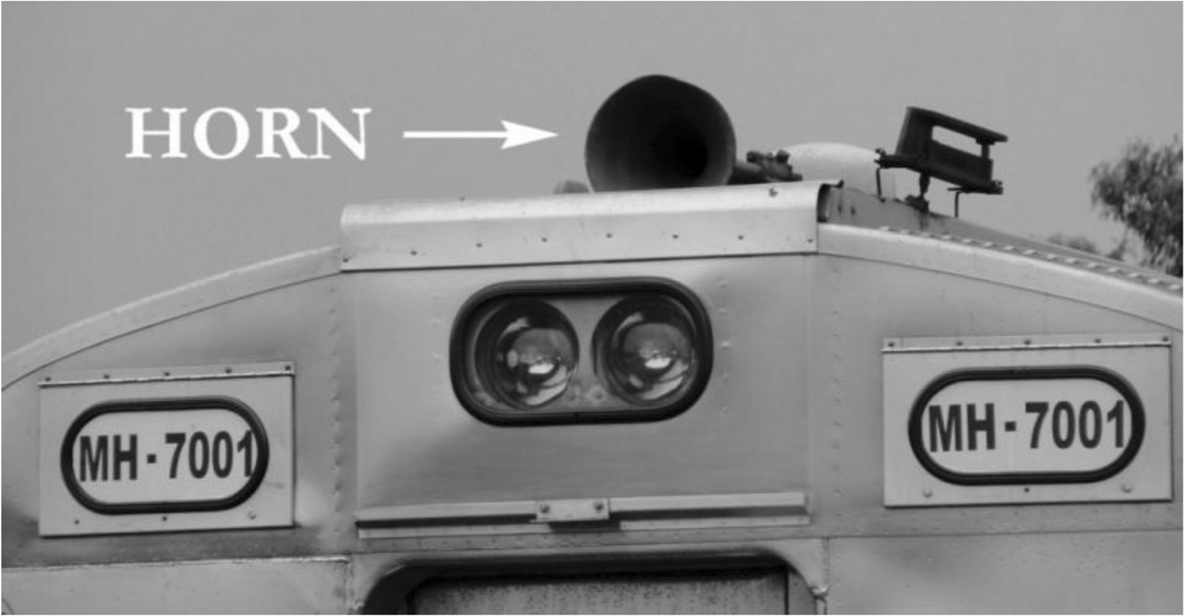
Photograph of a train horn mounted on the roof of the locomotive.

**Figure 4:**
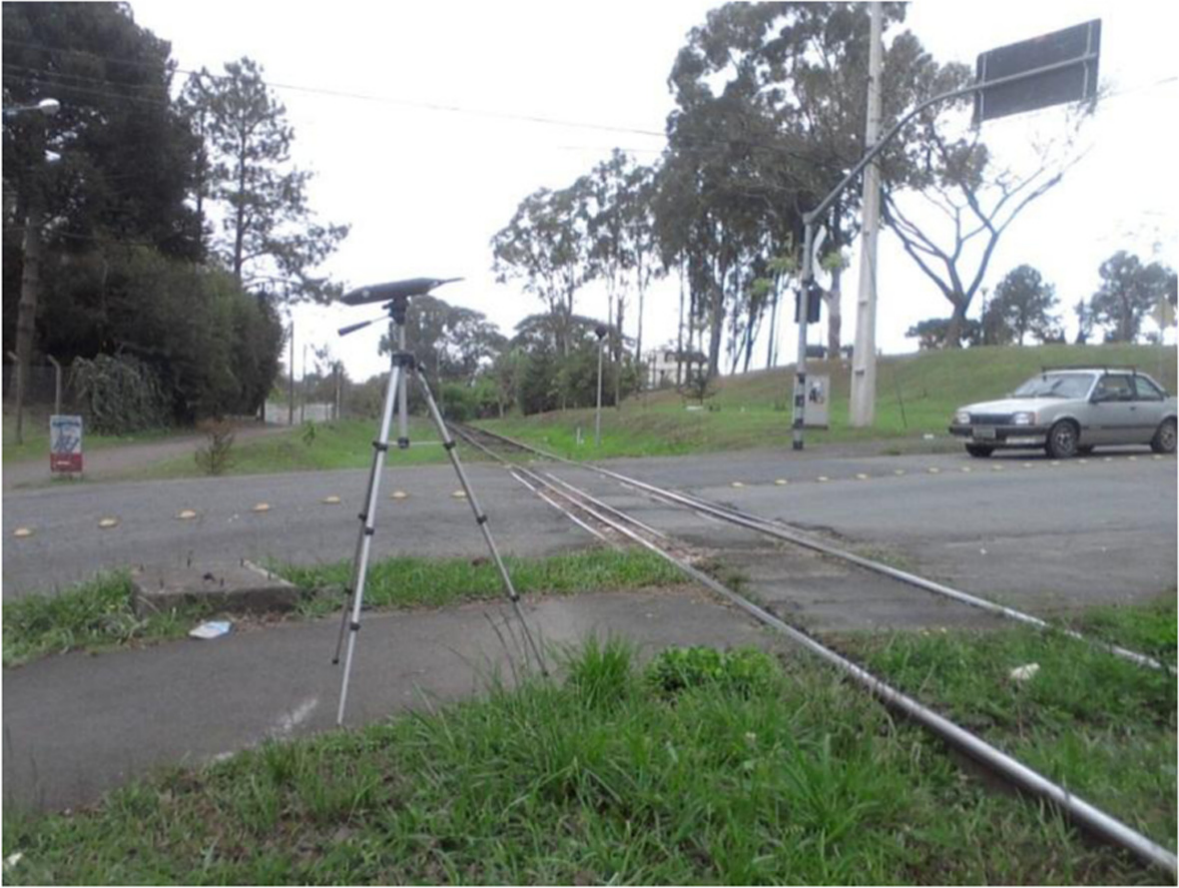
Urban street railroad crossing without safety barriers.

**Figure 5:**
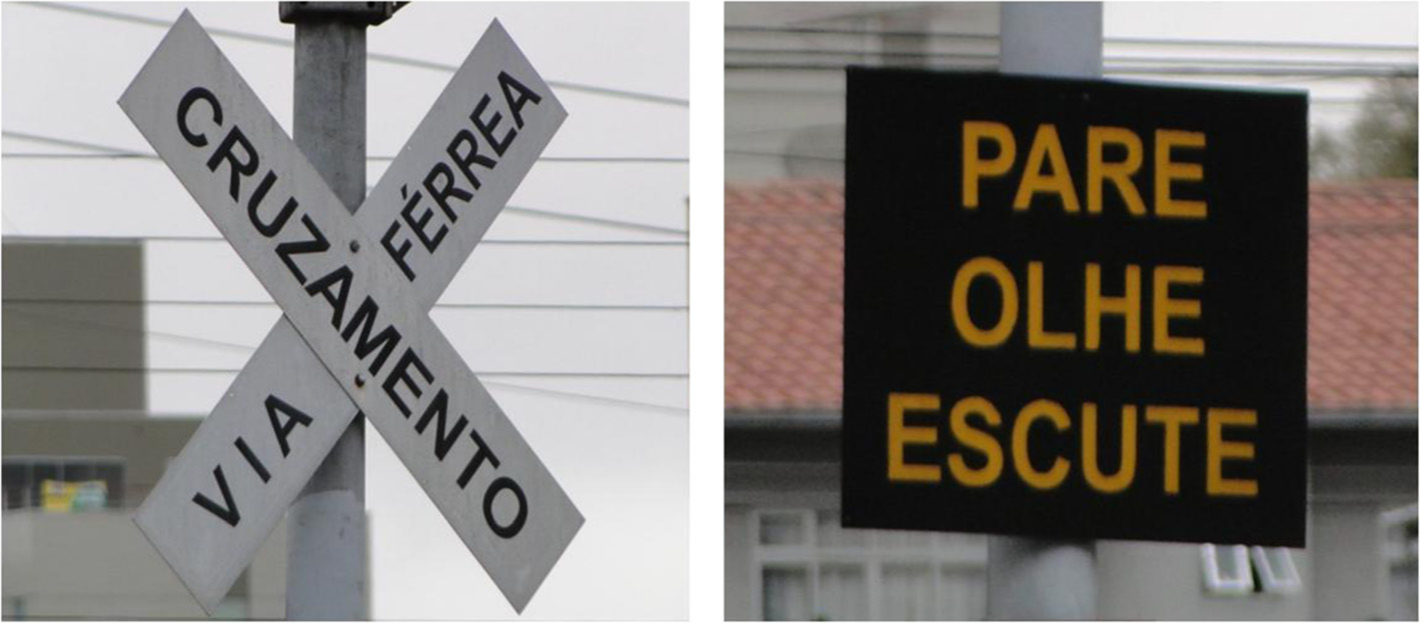
**Safety signs warning of urban street railway crossings.** Left: RAILWAY/CROSSING; Right: warning sign, from top to bottom: STOP/LOOK/LISTEN.

On its route through the city, the railway line crosses urban thoroughfares and passes through residential neighborhoods. As a safety measure, trains blow their horn before they reach a railroad crossing (see Figure 3). However, there are no barriers that close automatically to prevent the passage of vehicles, and fatal accidents are not infrequent. Figure 4 shows a typical railroad crossing without barriers in Curitiba, unlike Germany, for instance, where they typically exist. Figure 5 shows safety signs drawing attention to railroad crossings. The trains pass through 40 crossings and blow their horn at least three times as they approach a crossing, thus blowing their horns at least 120 times as they pass through the city. Since an average of ten trains pass through the city each day, their horns are blown at least 1200 times per day.

The railway noise is a serious environmental problem, as reported in the lengthy study by Fields and Walker [22]. These authors evaluated the response to railway noise in residential areas in Great Britain, and reached the following conclusion: “*Noise is rated as the most serious environmental nuisance caused by railways*.” The literature on environmental noise pollution contains several reports on railway noise in different countries, including the United Kingdom, France, Japan, Sweden, the Netherlands, the United States of America, Switzerland, and Germany [16,22,31-36]. In Brazil, however, studies about railway noise are as rare as to be practically nonexistent, with a very exceptions such as the works of Bertolli and de Paiva [37] and Roland and Zannin [38].

This paper describes an assessment of the annoyance caused by railway noise in a large Latin American city, based on noise measurements, noise mapping, and interviews.

## Materials and methods

The environmental impact generated by railway noise in the city of Curitiba was characterized based on several parameters: 1) noise level measurements at railroad crossings with the train horn blowing; 2) noise level measurements at railroad crossings without the train horn blowing; 3) noise maps showing the situation of noise pollution generated by train horn blowing; 4) noise maps without train horn blowing; 5) noise measurement at the receiver, i.e., inside the home of a resident in a neighborhood affected by railroad noise; and 6) interviews with the population of a district through which the railway runs.

The noise levels – equivalent sound pressure levels, L_eq_ - were measured according to the Brazilian standard for noise assessment in urban environments, NBR 10151 [39], at various points along the railway line. In addition to the L_eq_, the maximum and minimum noise levels were measured. A Brüel and Kjaer 4231 sound calibrator and five Type 1 integrating sound pressure level (SPL) meters (Brüel and Kjaer B&K 2270, B&K 2260 (two of this model), B&K 2250 and B&K 2238) were used for the noise measurements.

Advances in computational resources have led to the development of several software programs for analyzing environmental noise pollution [40]. The SoundPLAN Version 6.2 software package was used in this study for the calculations involved in noise mapping to evaluate the noise levels caused by the railway. The current literature contains several studies which used noise mapping as a tool for environmental impact assessment (see, for instance [41-44].

The German prediction method for railway noise, *Schall* 03, was used to calculate the noise generated by trains [34,45]. In this method, the Mean Emission Level – MEL can be calculated in two ways: 1) From the data flow, and 2) From data entered directly into the software, e.g., noise measurements [45]. In this study, noise mapping was performed by entering the measured noise levels as input data in the software. After entering this data, specific corrections must be made for the MEL, considering, among other factors, type of track, bridges, and railroad crossings.

To simulate the noise levels emitted by train horns, measurements were taken *in situ*, to enter them as input data into the software. After entering the railway data into the software SoundPlan, an area of calculation must be chosen with a given certain grid (average number of calculation points). For an environment that is little urbanized, a grid spacing of 20 to 50 meters suffices for acoustic mapping. However, for a highly urbanized region, the handbook of the software SoundPlan, indicate that the grid spacing may vary from 5 to 15 meters. The grid adopted in this work was 5x5 meters in order to produce a higher level of detail of the noise levels on the acoustic map. The height of the grid used in the calculation, as well as by other authors was 4 meters.

The simulated data were calibrated by placing a receiver point at the site where each noise measurement was taken. Measured and simulated levels were compared at the same height, in this specific case, 1.2 m. The calibration was based on the recommendations of the European Commission Working Group – Assessment of Exposure to Noise [46], for which the expected uncertainty is 4.6 dB(A) [47] when measured and simulated values are compared.

**Figure 6:**
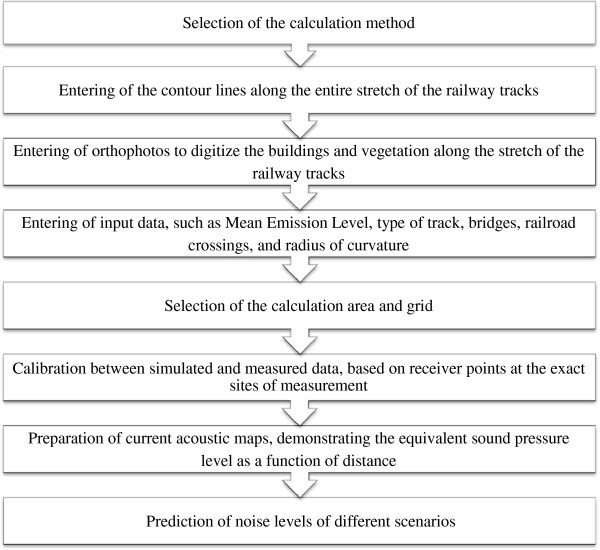
Steps involved in the computer simulations.

The steps taken to simulate rail noise are shown in the flow diagram shown in Figure 6.

To assess the degree of annoyance due to noise generated by the train traffic, interviews were conducted with the residents of neighborhoods that are crossed by the railway line. The researchers handed a questionnaire to each household. One person per household responded to the questionnaire. After two weeks the researchers collected the questionnaires. One hundred and fifty questionnaires were distributed, and 130 were collected. This research was performed according to the Helsinki Declaration.

## Results and discussions

The trains passing though the city of Curitiba follow a pattern that is repeated at each railroad crossing. Shortly before reaching each crossing, the train blows its horn three times. Ten railroad crossings were evaluated, and noise measurements were taken at each of them in three different situations: A) Train passing with horn blowing, B) Train passing without horn blowing, and C) Surroundings of the railroad crossing without the presence of the train.

**Figure 7:**
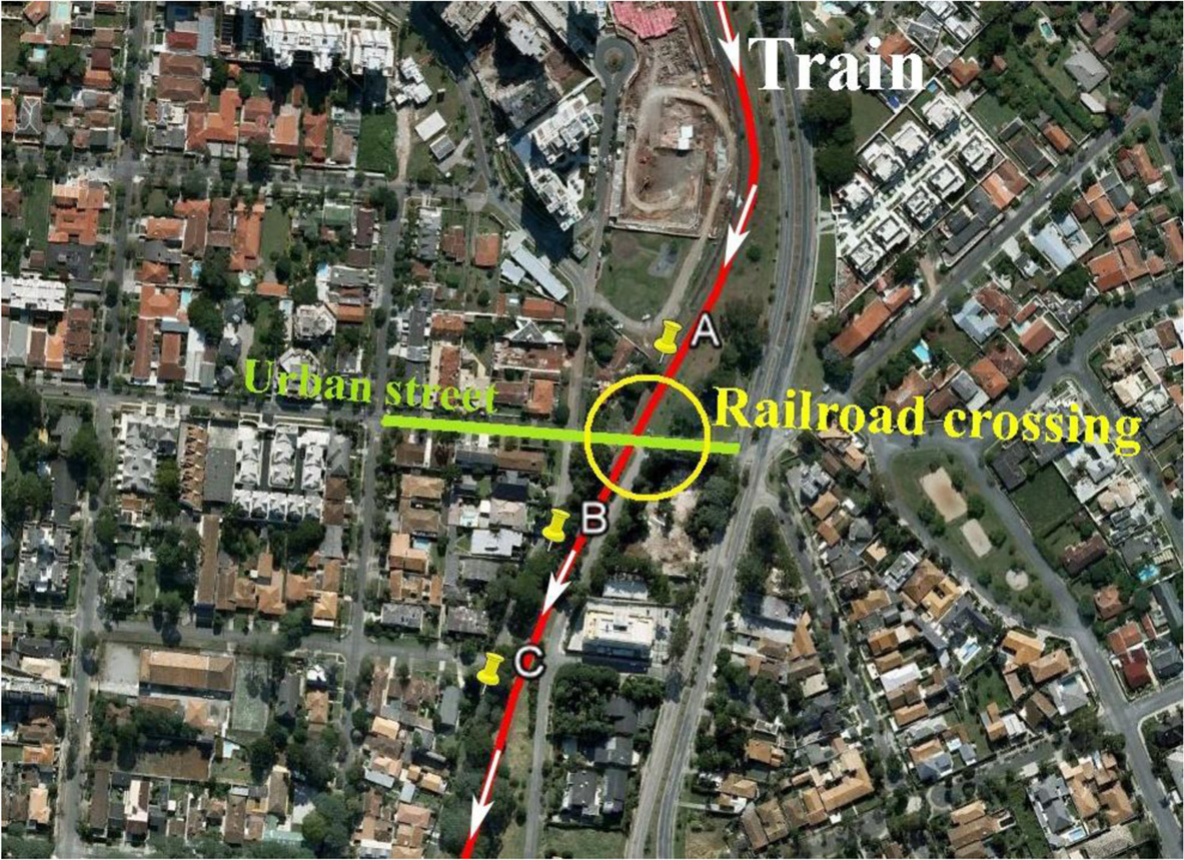
Measurement points along the railway line: A – Train passing with horn blowing, B – Train passing without horn blowing, C – Ambient noise without train passing.

Figure 7 shows an example of a railroad crossing where a set of measurements were taken along the railroad, as described above. Each railroad crossing was assigned a number from 1 to 10, and the three different measurement situations were assigned a subindex (A, B, and C).

Table 1 describes the noise level measurements at various points along the railway line, for the situations described in Figure 7.

**Table 1:** Noise levels measured along the railroad and surroundings

**Measurement site - Railway crossing -**	**Measurement situation**	**Characterization of the measurement**	**L**_**min **_**dB(A)**	**L**_**eq **_**dB(A)**	**L**_**max **_**dB(A)**
1	A	Train passing with horn blowing	90.1	108.3	121.4
	B	Train passing without horn blowing	62.4	79.8	91.0
	C	Ambient noise without train passing	48.3	59.9	76.2
2	A	Train passing with horn blowing	71.3	101.0	108.3
	B	Train passing without horn blowing	49.9	79.9	91.4
	C	Ambient noise without train passing	41.9	53.9	75.7
3	A	Train passing with horn blowing	74.5	109.8	115.6
	B	Train passing without horn blowing	60.0	80.0	93.4
	C	Ambient noise without train passing	46.5	59.4	78.6
4	A	Train passing with horn blowing	69.4	102.7	109.7
	B	Train passing without horn blowing	66.2	87.4	95.7
	C	Ambient noise without train passing	43.1	53.6	72.9
5	A	Train passing with horn blowing	86.2	108.9	115.1
	B	Train passing without horn blowing	66.5	84.3	90.9
	C	Ambient noise without train passing	50.3	57.9	73.0
6	A	Train passing with horn blowing	77.8	108.9	115.6
	B	Train passing without horn blowing	77.1	82.5	89.4
	C	Ambient noise without train passing	45.7	59.7	84.7
7	A	Train passing with horn blowing	61.1	100.0	109.9
	B	Train passing without horn blowing	71.6	80.8	87.0
	C	Ambient noise without train passing	55.0	70.5	87.0
8	A	Train passing with horn blowing	78.5	108.1	116.5
	B	Train passing without horn blowing	73.6	81.6	90.9
	C	Ambient noise without train passing	54.6	65.7	80.4
9	A	Train passing with horn blowing	67.2	105.9	112.6
	B	Train passing without horn blowing	64.9	82.3	93.4
	C	Ambient noise without train passing	51.4	62.7	86.4
10	A	Train passing with horn blowing	53.8	95.0	111.4
	B	Train passing without horn blowing	67.1	77.5	93.4
	C	Ambient noise without train passing	51.9	60.6	75.6

The simulated data were calibrated by placing a receiver point at the site where each noise measurement was taken. Measured and simulated noise levels were compared at the same height, in this specific case, 1.2 m.

The calibration was based on the recommendations of Licitra and Memoli [47], whereby it is expected that the difference between the simulated and measured noise level does not exceed the value of 4.6 dB (A).

Based on the above, Table 2 shows the measured noise levels and the noise levels calculated by the software SoundPLAN. As can be seen in Table 2, last column to the right, the differences between simulated and measured values was below 4.6 dB (A), as recommended by Licitra and Memoli [47].

**Table 2:** Comparison of measured and simulated noise levels

**Measurement site along the railway lines**	**Measurement situation**	**Measured L**_**eq **_**dB(A)**	**Simulated L**_**eq **_**dB(A)**	**Difference between measured and simulated noise**
1	A	108.3	107.5	0,8
	B	79.8	80.3	−0,5
2	A	101.0	100.2	0,8
	B	79.9	79.3	0,6
3	A	109.8	111.1	−1,3
	B	80.0	81.5	−1,5
4	A	102.7	103.2	−0,5
	B	87.4	88.2	−0,8
5	A	108.9	108.3	0,6
	B	84.3	84.7	−0,4
6	A	108.9	108.4	0,5
	B	82.5	83.1	−0,6
7	A	100.0	100.7	−0,7
	B	80.8	82.8	−2,0
8	A	108.1	109.2	−1,1
	B	81.6	83.3	−1,7
9	A	105.9	104.3	1,6
	B	82.3	82.9	−0,6
10	A	95.0	95.6	−0,6
	B	77.5	76.9	0,6

The railroad crossings listed in Table 1 are located in Urban Residential Areas for which Law 10625 of the municipality of Curitiba [48], which enacts laws about urban noise, establishes that daytime noise levels, from 7:01 a.m. to 7:00 p.m., should not exceed 55 dB(A). Thus, it is evident that the noise generated by passing trains exceeds the limits established by municipal legislation, resulting in noise pollution.

**Figure 8:**
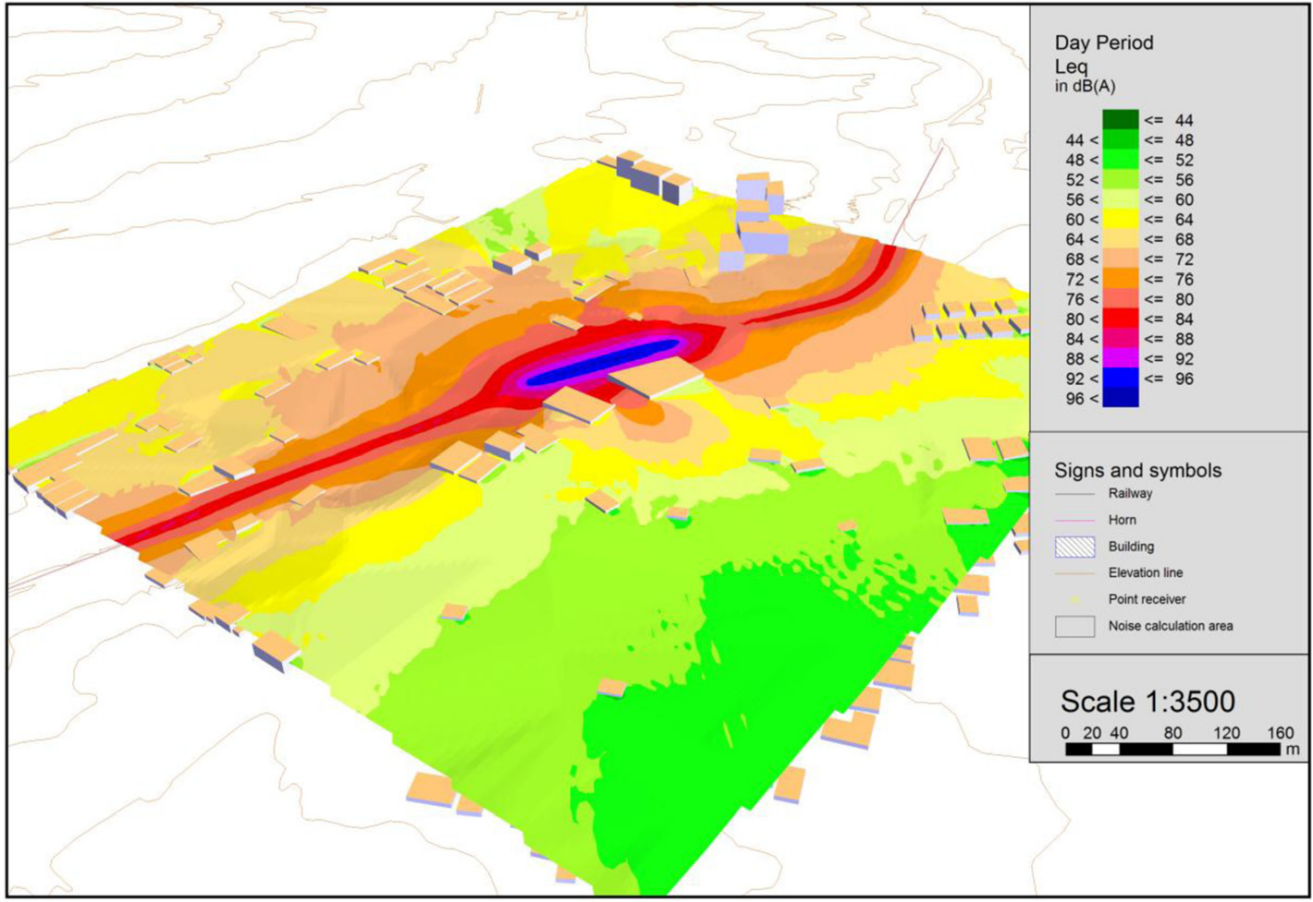
3D noise map of the situation when the train is blowing its horn.

To analyze the noise generated by rail traffic based not only on measurements, SoundPLAN software was used to calculate noise maps for two situations: 1) Train passing with horn blowing, 2) Train passing without horn blowing. The results obtained from these simulations indicate how high the noise levels are. Figure 8 show the noise map in three dimensions, of when a train passes with its horn blowing.

The map in the above figure show that the passage of trains blowing their horns generates noise levels of 80 to 92 dB(A) at the facades of the homes closest to the railway line. Moreover, they indicate that the noise levels that reach the more distant homes range from 68 to 80 dB(A). Figure 8 also indicates that together with the train, the noise levels at the centerline of the noise map exceed 96 dB(A). The noise maps were calculated based on railroad crossing no. 2 and measurement situation “A,” as indicated in Tables 1 and 2.

Curitiba’s urban legislation [48] establishes a maximum daytime noise level of 55 dB(A) for the area of this study, which is a residential area. Therefore, the situation is clearly one of noise pollution, since the noise levels generated far exceed the legally established limit. It should be kept in mind, as explained earlier, that trains pass through the city about ten times a day, blowing their horns about 1200 times as they approach the city’s 40 railroad crossings.

**Figure 9:**
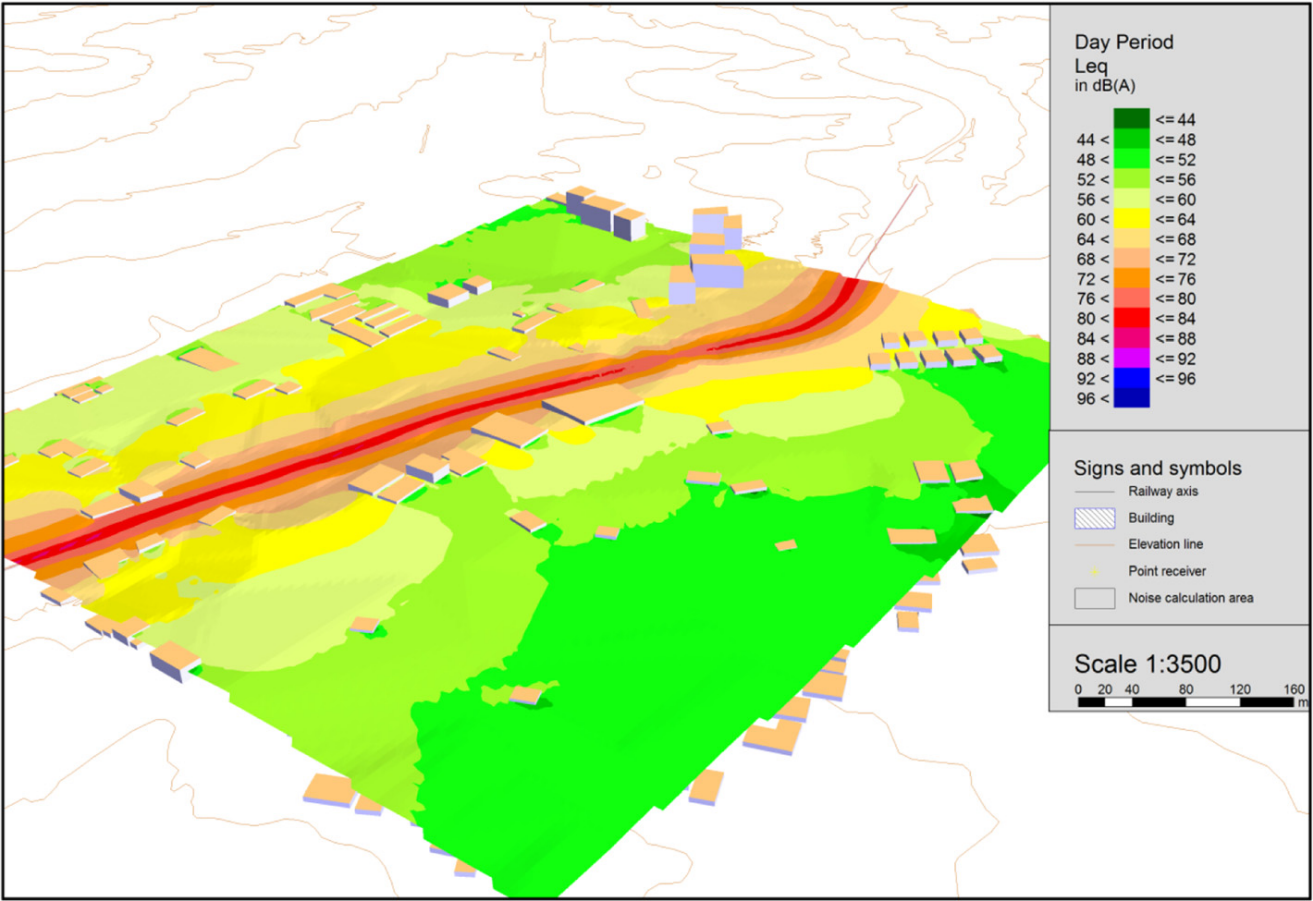
3D noise map of the train passing without blowing its horn.

The map in Figure 9, show the scenario when the train does not blow its horn. The noise emission level decreases significantly with the elimination of the blowing horn. The noise levels in the proximities of the rail line vary from 68 to 80 dB(A), in contrast with the situation with the horn blowing, when the levels varied from 80 to 92 dB(A).

The noise maps indicate the efficiency of this noise control measure, the elimination of the blowing horn. However, it is also clear that although the noise levels are drastically reduced, they still exceed the noise limits established by municipal legislation.

The analysis of questionnaires filled out by residents indicated that they are well aware of the problem of noise generated by the trains, since the majority, 62%, have lived there for one to five years, and 25% have lived there for over five 5 years. Only 18% of the respondents have lived there for less than a year.

Residents were asked to assess whether – *during the time they have lived there* – the noise has increased, remained the same or decreased. Among the respondents, 65% indicated that the noise has increased, 33% indicated that the noise has remained the same, and only 2% stated that it has decreased. With regard to the intensity of noise, 57% classified it as very intense, 35% as intense, and 8% as little intense.

When asked if the noise in the neighborhood bothers them, 84% answered *YES*, 15% answered *NO*, and 1% did not answer the question. Asked if they believe that environmental noise is harmful to their health, 98% of the residents answered *YES* and only 2% answered *NO*.

Residents were asked whether – *they find the noise irritating*, to which 92% answered *YES* and 8% *NO*. Table 3 lists the noises considered sources of irritation to residents that answered *YES* to the question: “Is this noise source a cause of irritation?”

**Table 3:** **Noise as a cause of *irritation *** **and percent of interviewees affected by it**

**Sources of noise that cause *irritation***	**Respondents that answered *yes***
Automotive vehicles	52%
***Trains***	***95***%
Churches and temples	2%
Fireworks	10%
Animals	9%
Toys and games	1%
Construction sites	34%
Noisy neighbors	26%
Nightclubs	3%
Others	6%

The residents were asked whether noise leads to – *poor concentration*, to which 86% said *YES*, 13% answered *NO*, and 1% did not respond. Table 4 lists the noise sources that interfere with concentration, for residents who answered *YES* when asked: “Does this source of noise lead to poor concentration?”

**Table 4:** **Noise causing *poor concentration *** **and percent of interviewees affected by it**

**Sources of noise that cause *poor concentration***	**Respondents that answered *yes***
Automotive vehicles	57%
***Trains***	***88***%
Churches and Temples	4%
Fireworks	13%
Animals	9%
Toys and games	4%
Construction sites	31%
Noisy neighbors	22%
Nightclubs	5%
Others	5%

Residents were asked whether the noise causes – *headache*, to which 59% responded *YES*, 39% answered *NO*, and 2% did not respond. Table 5 lists the noise sources causing headaches in residents who answered *YES* to the question: “Does noise give you headaches?”

**Table 5:** **Noise as a cause of *headaches *** **and percent of interviewees affected by it**

**Sources of noise that cause *headaches***	**Respondents that answered *yes***
Automotive vehicles	53%
***Trains***	***96***%
Churches and temples	3%
Fireworks	6%
Animals	6%
Toys and games	3%
Construction sites	23%
Noisy neighbors	18%
Nightclubs	4%
Others	3%

The residents were asked what time of the day they consider the most bothersome in terms of noise. The great majority, 88%, stated that the most bothersome time is the nighttime. Asked if the noise causes them – *insomnia*, 73% of the respondents answered *YES*, and 27% *NO*.

The respondents who answered *YES* when asked whether noise caused insomnia were asked to point out the main sources causing insomnia. Table 6 lists the noise sources that cause insomnia in residents who responded *YES* to the question: “Does noise interfere in your sleep?”

**Table 6:** **Noise causing *insomnia *** **and percentage of respondents affected by it**

**Sources of noise causing *insomnia***	**Respondents that answered *yes***
Automotive vehicles	61%
***Trains***	***100***%
Churches and temples	1%
Fireworks	11%
Animals	9%
Toys and games	1%
Construction sites	29%
Noisy neighbors	25%
Nightclubs	4%
Others	4%

The interviewees were asked how frequently – *their sleep is disrupted by noise*, to which 58% answered *Often*, 32% *Sometimes*, 9% *Never*, and 1% did not answer. They were then asked whether – *sleep is interrupted by the noise of the train*, with 70% claiming that their sleep is *Interrupted Frequently*, 21% *Sometimes* 8% *Rarely or Never*, and 1% did not answer.

Table 7 lists the times of the day when, according to the residents, the noise of the train is the most frequent nuisance.

**Table 7:** **Time of the day when the train**’**s noise is the most annoying and percentage of respondents affected by it**

**Time when *sleep is interrupted *** **by train noise**	**Percentage of respondents**
12 – 2 a.m.	18%
2 – 4 a.m.	18%
***4*** – ***6 *** **a**.**m**.	***37***%
***6*** – ***8 *** **a**.**m**.	***43***%
8 – 10 a.m.	19%
10 a.m. – 12 p.m.	2%
12 – 2 p.m.	1%
2 – 4 p.m.	0%
4 – 6 p.m.	0%
6 - 8 p.m.	3%
8 – 10 p.m.	9%
***10 p***. ***m***. – ***12 a***. ***m***.	***35***%

**Figure 10:**
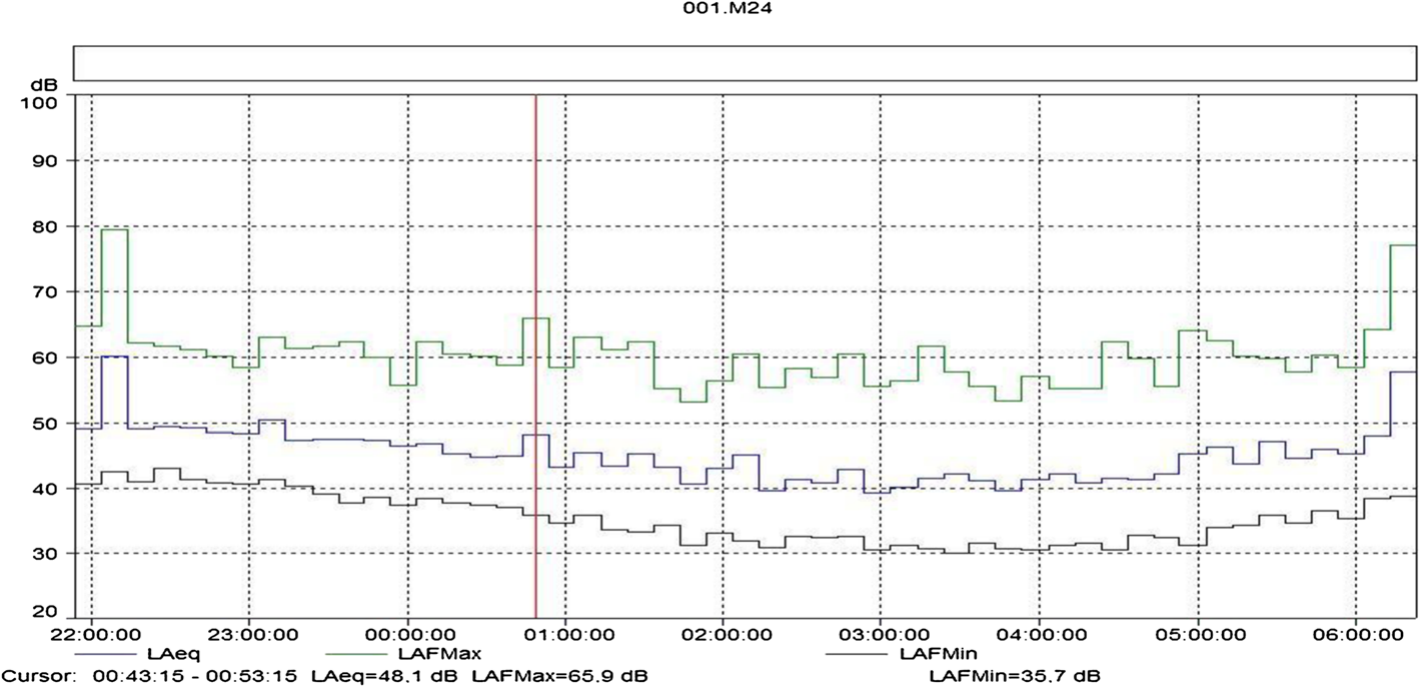
**Noise levels as a function of the time of day.** Nighttime measurements of train noise taken at the home of a resident.

As reported above, 88% of the interviewees indicated that the noise of the train is the most annoying during the nighttime. In view of this finding, measurements were taken of the nighttime noise generated by passing trains. To this end, a sound level meter was installed in a sound receiving location – the home of a resident. The distance from the railway tracks to the receiver site (the resident’s home) is about 200 meters. As Figure 10 shows, the measurements started before 10 p.m. and ended after 6 a.m. A B&K 2238 sound level meter was used and the measurements were taken with a *datalog* module (noise levels vs. time of measurement), with measurements recorded at 10 minute intervals.

Figure 10 indicates that two trains passed by the measurement location between 10 p.m. and 6 a.m., one at 10:10 p.m. and the other at 6:20 a.m. Both trains blew their horn, as evidenced by the high values of maximum sound level, L_max_, and equivalent sound level, L_eq_.

Figure 10 shows how high the noise level is when a train passes with its horn blowing, since the maximum sound levels reached nearly 80 dB(A) at the railroad crossing at 10:10 p.m. and 78 dB(A) at 6:20 a.m. The equivalent sound pressure level reached L_eq_ = 60 dB(A) at 10:10 p.m. and L_eq_ = 58 dB(A) at 6:20 a.m.

Curitiba’s municipal Law 10625, which regulates noise in communities [49], establishes that the noise levels, L_eq_, from 10 p.m. to 7 a.m. cannot exceed 45 dB(A) in the region where the nighttime measurements were taken. Therefore, it is a clear violation of this law during the nighttime.

The measurement shown in Figure 10 proves what the residents claimed, as indicated in Table 7, i.e., the daytime periods from 4 to 6 a.m. and 6 to 8 a.m., and the nighttime period from 10 p.m. to midnight are the periods of greatest annoyance due to train noise. 37% of the respondents stated that the noise between 4 and 6 a.m. was the most annoying, while 43% stated it was between 6 and 8 a.m., and 35% claimed that the noise between 10 p.m. and midnight was the most disruptive.

Lastly, the residents were asked whether they believe that local noise can devalue their home, to which 69% responded *YES*, 28% *NO*, and 3% did not answer the question.

Evaluating the effect of aircraft noise on home value depreciation, Espey and Lopez [50] showed that the value of homes located in areas close to an airport, where noise levels were 65 dB(A) or higher, was about $ 2400 lower than similar homes located in areas not considered noisy. Railway noise also has an impact on the value of homes. The train horn is considered a major cause of high noise levels near railway lines. Bellinger [49] evaluated the cost of noise generated by blowing train horns in a small town in Pennsylvania. According to him, real estate market values depreciate by 4.1% for every 10 dB above the background noise level. Considering the 256 homes affected, the losses represented a total of about $ 4 million in 2004 market values.

## Conclusions

The present study evaluated the noise generated by railway in a large Latin American city. Several analytical techniques were used – measurements of noise levels during the passage of the train with and without its horn blowing, measurement of noise levels in the home of a resident affected by the noise of the train and calculation of noise mapping. Lastly, to assess the degree of annoyance due to noise generated by train, interviews were conducted with the residents of neighborhoods that are crossed by the railway line.

As in the study presented here, research conducted in Poland by Szwarc et al. [51], and in Germany by Czolbe [52] also used noise maps to diagnose the impact of noise generated by railway traffic in urban areas.

The measurements indicated that the noise levels generated as the train passes with its horn blowing are extremely high, clearly violating Curitiba’s noise legislation. The noise mappings showed that a simple solution to control noise would be for the trains to pass through the city without blowing their horns. However, although the noise levels are significantly lower when the train’s horn is not blown, they still exceed the levels established by municipal legislation.

The city has been suffering from this problem for decades. The solution to the problem would be to remove the railway line passing through the city. However, lack of resources and of political will are two obstacles to the removal of the trains passing through residential areas within the city.

The residents were found to feel strongly affected by noise generated by passing trains. Train noise causes *irritation and annoyance*, *headache*s, *poor concentration* and *insomnia*. In terms of noise pollution, 88% of the respondents cited nighttime as the most critical time of the day. As shown in this paper, the research of Fields and Walker [22] in Great Britain, Lambert et al. [16] in France, and Ali [53] in Egypt also show that the population neighboring railways feels disturbed by the noise of the train.

This study showed that the vast majority of residents surveyed (69%), believe that the noise of the train can devalue their property. We would do well to keep in mind the words of Fields and Walker [22]: “*Noise is rated as the most serious environmental nuisance caused by railways*.”

## Competing interests

The authors declare that they have no competing interests.

## Authors’ contributions

Both authors participated in the planning and performance of the measurements and simulations. Both authors contributed equally to the writing of the manuscript. Both authors read and approved the final manuscript.

## Aknowledgments

The authors gratefully acknowledge the German Government, through the German Academic Exchange Service – DAAD (Deutscher Akademischer Austauschdienst) and the Brazilian Government, through the National Council for Scientific and Technological Development – CNPq, for their financial support, which enabled the purchase of the sound level meters and software used in this study. The authors would like to thank the reviewers who contributed to the improvement of this work with their observations.
